# Effect of nifedipine with and without sildenafil citrate for the management of preterm labor in pregnant women: A randomized clinical trial

**DOI:** 10.18502/ijrm.v21i5.13471

**Published:** 2023-05-12

**Authors:** Shahla Nasrolahei, Seyedeh Arezoo Hoseini, Seyedeh Azadeh Hosseini, Seyedeh Narjes Khatoon Hosseini, Seyedeh Sahar Hosseini, Parsa Moradian Lotfi

**Affiliations:** ^1^Department of Obstetrics and Gynecology, School of Medicine Endometrium and Endometriosis Research Center, Fatemieh Hospital, Hamadan University of Medical Sciences, Hamadan, Iran.; ^2^Department of Obstetrics and Gynecology, Zanjan University of Medical Sciences, Zanjan, Iran.; ^3^Iran University of Medical Sciences, Tehran, Iran.; ^4^Bahçeşehir Medical University Istanbul Turkey, Istanbul, Turkey.; ^5^Hamadan University of Medical Sciences, Hamadan, Iran.

**Keywords:** Nifedipine, preterm labor, Sildenafil citrate, Randomized trial.

## Abstract

**Background:**

Preterm labor is one of the main causes of neonatal mortality and its treatment is still challenging.

**Objective:**

The study aimed to compare the effectiveness of nifedipine (Nif) with and without sildenafil citrate (SC) for the treatment of preterm labor in pregnant women.

**Materials and Methods:**

In this clinical trial study, 126 pregnant women referred to the Fatemieh hospital, Hamadan, Iran with a complaint of preterm labor were evaluated. Participants were randomly divided into 2 groups of Nif 20 mg orally (single dose), then 10 mg every 6-hr, and at the same time vaginal SC 25 mg every 8 hr (Nif + SC) or Nif alone. Treatment was continued for 48-72 hr if uterine contractions did not resolve in both groups. Delivery rates at the time of hospitalization and neonatal outcome were compared between the 2 groups.

**Results:**

No statistically significant difference was observed between the 2 study groups in terms of mean age, gestational age, body mass index, and parity. 76.2% of Nif + SC participants in the first 72 hr of hospitalization and 57.2% of Nif participants remained without delivery (p = 0.02). The neonatal hospitalization rate of the Nif + SC group in the neonatal intensive care unit was 25.4% and in the Nif group was 42.9% (p = 0.03).

**Conclusion:**

Nif with SC is superior to Nif alone in women at risk of preterm labor due to increasing gestational age and better neonatal outcomes.

## 1. Introduction 

Preterm labor (PTL) affects about 6-7% of pregnancies in developed countries and is responsible for about two-thirds of non-anomaly neonatal deaths as well as long-term complications of fetal prematurity (1). Risk factors for labor include infection, maternal-fetal stress, uterine dilatation, and one of the most important risk factors includes a previous history of delivery (2). Newborns born before 37 wk suffer various morbidities, largely due to organ system immaturity, such as pulmonary (respiratory distress syndrome), gastrointestinal (necrotizing entrocolitis), immunological (perinatal infection), central nervous system, ophthalmological, cardiovascular, renal hematological, and endocrinological. Over the past 2 decades, no progress has been observed in prevention and treatment of delivery, in order to reduce the prevalence of delivery in developed countries acquired only in the treatment category (3).

Numerous tocolytic factors are used to stop uterine contractions (4). Magnesium sulfate is the tocolytic used in plenty in our region (5). However, according to recent studies, this drug is known for its common maternal and fetal complications and the existence of contradictory reports about its effectiveness and cost. One of them is a drug from the group of calcium channel blockers called nifedipine (Nif) (6). Calcium channel blockers like Nif through various mechanisms inhibits calcium entrance using channels in the cell membrane. Nif has been more effective and safer than Ritodrine and other calcium channel blockers in treating PTL. In other meta-analysis, Nif has been suggested as the drug of choice for preventing PTL (7).

Sildenafil citrate (SC) is a drug used in cases of erectile dysfunction, Pulmonary hypertension, Raynaud's phenomenon (8). It has also been used in studies of intrauterine growth restriction and PTL (9). SC has been listed based on Food and Drug Administration in category B in pregnancy. SC inhibits the enzyme phosphodiesterase type 5. Sildenafil, by selectively inhibiting phosphodiesterase type 5, increases the cyclic concentration of guanosine monophosphate in vascular smooth muscle and also increases smooth muscle expansion (10).

Myometrium expresses several ion channels, the most abundant of which is the activated potassium channel, which plays an important role in uterine excitability. Although tocolytic factors do not cause significant prolongation of pregnancy, but in some women, they can delay delivery by at least 48 hr. This break provides enough time for corticosteroid treatment to mature the fetal lung (11).

Due to the limited study on the effects of Nif and sildenafil (9, 12, 13), this clinical trial study focused on the effects of Nif and sildenafil or Nif alone in women at risk for PTL.

## 2. Materials and Methods 

**Figure 1:**
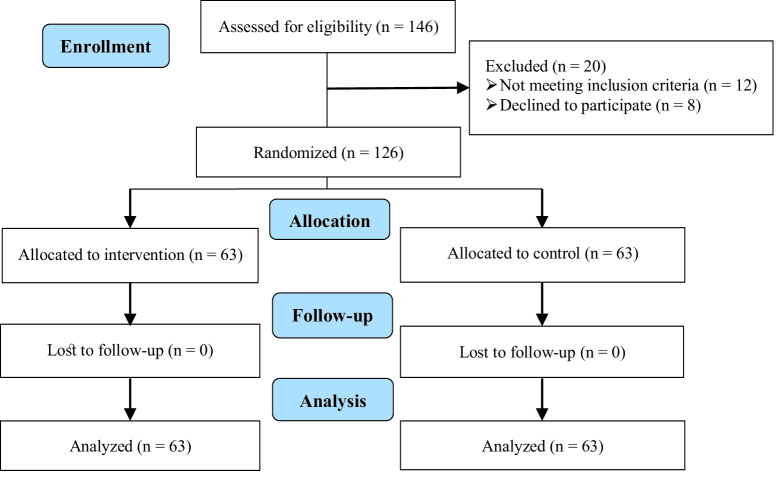
Diagram of the study design.

This clinical trial study was performed from February 2021 to May 2022. The target population in this study were pregnant women referring to Fatemieh hospital in Hamadan (west of Iran). PTL is any delivery after 20 wk and before the completion of 37 wk gestation. Figure 1 shows how participants were selected and followed; initially 146 women were assessed for eligibility of which 20 women were excluded for not meeting the inclusion criteria or declined to participate, finally 126 participants were randomized for intervention. Before entering the study, written consent was obtained from the women participating in the study. Inclusion criteria were: PTL at the age of 15-45 yr, cases of threatened PTL (uterine contractions > 4 in 20 min with cervical dilatation and effacement), between 24-34 wk gestation, intact fetal membranes, cervical dilatation less than 4 cm, no major chronic medical disorder, had no contraindication for Nif and SC therapy, and had no previous history of PTL. Exclusion criteria were multiple pregnancy, suspected chorioamnionitis, unexplained fetal tachycardia or maternal temperature > 380 C, cervical dilatation > 4 cm, ruptured fetal membranes, chronic diseases (hypertension, renal failure, diabetes), contraindication of Nif or SC (kidney or liver or heart disease, inflammatory bowel disease, severe hypotension), previous history of PTL, and general contraindications to tocolytic therapy such as intrauterine fetal demise, sever preeclampsia, and eclampsia.

One group of women was given Nif tablet (manufactured by Zahravi Pharmaceutical Co.) and the other group was given Nif with SC (manufactured by Pars Darou Pharmaceutical Co.) to prevent PTL. One group received Nif tablet 20 mg single dose and then 10 mg every 6-8 hr, at the same time vaginal sildenafil 25 mg tablet every 8 hr and in the opposite group, Nif alone (13). In both groups women received standard corticosteroid therapy to accelerate fetal lung maturation (betamethasone 12 mg intramuscularly and repetition of the same dose 24 hr later). Treatment was repeated for 48-72 hr if uterine contractions did not resolve. Maternal pulse, blood pressure, and uterine contractions were recorded every 15-30 min for 4 hr in time of prescribing drugs. If uterine contractions are resolved, participants could be discharged after 24 hr with a progesterone suppository to prevent preterm delivery. Gestational age at the time of delivery, neonatal weight, and neonatal hospitalization in the intensive care unit were the secondary outcomesof this study. These outcomes were obtained from patient's medical record information or examination.

### Randomization and blinding

In this study, randomization was performed by block randomization (block, n = 4). Participants and the person evaluating the patient outcome were unaware of participants being assigned to the intervention groups. The study conducted was double blinded; the collected data were coded into the statistical software, which was unknown to the statistical analyst of the groups.

Data were entered in SPSS 16 software version (SPSS Inc. Chicago, IL, The USA) and analyzed. To express quantitative descriptive findings, mean and standard deviation, and for qualitative findings, frequency and percentage were used. The Kolmogorov-Smirnov test was used to evaluate the normality of quantitative data, *t* test for comparison quantitative variables, and Chi-square for qualitative variables. The sample size was calculated according to findings of the previous study (*μ* ${}_{1}$ = 1.6, *μ* ${}_{2}$ = 1.7, σ ${}_{1}$ = 0.2, and σ ${}_{2}$ = 0.2) with 80% power and alpha equal 0.05 (13). 


$$n=\frac{{\left(Z1-\frac{\alpha }{2}+Z1-\beta \right)}^{2}\left(\sigma^{2}+\sigma^{2}\right)}{\left(\mu 1-\mu 2\right)\wedge 2}$$

### Ethical considerations

The Ethics Committee of Hamadan University of Medical Sciences, Hamadan, Iran (Code: IR.UMSHA.REC.1399.484) approved the study. Before intervention, informed consent were obtained from all participants.

## 3. Results 

In this clinical trial study, 126 pregnant women who met our inclusion criteria were randomly assigned into 2 groups (n = 63/each). No statistically significant difference was observed between the 2 study groups in terms of mean age of women (p = 0.61), gestational age (p = 0.07), and body mass index (p = 0.833). Approximately 20% of women experienced the first pregnancies without statistically significant differences between the 2 groups (p = 0.82) (Table I). In group Nif + SC 15 women (23.8%) and in group Nif 20 women (31.7) received a repeat dose (p = 0.32).

Infants whose mothers received Nif + SC had a higher difference in birth weight. These neonates had better outcomes so that the rate of hospitalization in the intensive care unit and respiratory disease were less with a statistically significant difference (p < 0.05). The infant mortality rates were lower in the combination treatment group but were not statistically significant (Table II).

As shown in table III, at the time of hospitalization in the first 72 hr after the intervention, the delivery rate in the Nif + SC group was lower than in the Nif group. 87.3% of the women in the Nif + SC group remained without delivery in the first 72 hr after the intervention and 79.4% in the Nif group remained without delivery (p = 0.23).

The frequency of gestational age was 28-32 wk at delivery with a statistically significant difference in the Nif group. While the rate of very severe preterm delivery was lower in the Nif + SC group and gestational age was more than 32 wk, it was not statistically significant (Table IV).

**Table 1:** Baseline characteristics in both groups


**Variable**		**Nif + SC**	**Nif**	**P-value**
Mean maternal age*		29.5 ± 5.9	28.9 ± 5.6	0.61
Mean gestation age*		29.2 ± 2.9	28.3 ± 2.8	0.07
BMI (kg/m^2^)*		27.4 ± 4.7	27.2 ± 3.5	0.78
Parity**				
	**Nulliparous**	13 (20.6)	12 (19.0)	
	**Multiparous**	50 (79.4)	51 (80.9)	0.82
*Data presented as Mean ± SD. *t* test, **Data presented as n (%). Chi-square test. BMI: Body mass index, Nif: Nifedipine, SC: Sildenafil				

**Table 2:** Comparisons neonatal outcome in both groups


**Variable**	**Nif + SC**	**Nif**	**P-value**	**OR (95% CI)**
Birth weight (gr)*	2167.3 ± 362.2	2008.1 ± 30.8	0.01 ${}^{\text{a}}$	-
Admitted to NICU**	16 (25.4)	27 (42.9)	0.03 ${}^{\text{b}}$	2.2 (1.1-4.7)
Poulmnary**	20 (31.7)	31 (49.2)	0.04 ${}^{\text{a}}$	2.2 (1.1-4.2)
Neonatal death**	4 (6.3)	6 (9.5)	0.74 ${}^{\text{b}}$	1.5 (0.4-5.8)
*Data presented as Mean ± SD. **Data presented as n (%). a: *t* test, b: Chi-square, Nif: Nifedipine, SC: Sildenafil, NICU: Neonatal intensive care unit, OR: Odds ratio, CI: Confidence interva				

**Table 3:** Maternal outcome between both groups


**Gestational age (wk)**	**Nif + SC**	**Nif**	**P-value**	**OR (95% CI)**
**< 28 **	3 (4.7)	5 (8.0)	0.53 ≠	1.8 (0.5-6.6)
**28-32**	7 (11.1)	12 (19.0)	0.03*	2.3 (1.1-5.0)
**> 32**	53 (84.2)	46 (73.0)	0.10*	0.5 (0.2-1.2)
Data presented as n (%), *Chi-square, ≠ : Fisher's exact, Nif: Nifedipine, SC: Sildenafil, OR: Odds ratio, CI: Confidence interval				

**Table 4:** Comparison of frequency of gestational age at delivery in 2 study groups


**Variable**	**Nif + SC**	**Nif**	**P-value**	**OR (95% CI)**
**Delivery in 24 hr**	3 (4.8)	5 (7.9) ≠	0.71	1.7 (0.4-7.5)
**Delivery in 48 hr**	3 (4.8)	4 (6.3) ≠	1.00	1.3 (0.3-6.3)
**Delivery in 72 hr**	2 (3.2)	4 (6.3) ≠	0.68	2.1 (0.4-11.7)
**Un delivery**	55 (87.3)	50 (79.4)*	0.23	0.6 (0.2-1.7)
Data presented as n (%). *Chi-square, ≠ Fisher's exact, Nif: Nifedipine, SC: Sildenafil, OR: Odds ratio, CI: Confidence interval				

## 4. Discussion 

The present clinical trial study was performed to evaluate the efficacy of Nif alone or in combination with SC in the treatment of PTL.

Based on the search of available sources and the knowledge of the authors, no study has been published to study the simultaneous effect of Nif with SC in the treatment of PTL in the Iranian patient population.

The findings of our study showed that the combination of Nif with SC reduces the chance of delivery by almost 2 times, during the hospitalization period and the hospitalization of infants in the intensive care unit by a statistically significant difference, because it is more effective than Nif alone. Our findings also show that Nif with SC increases gestational age in women at the risk of PTL, although it was not statistically significant, which may be affected by sample size, it may also be clinically significant.

Currently, one of the remaining problems in the field of midwifery and infants is preterm birth. Preterm birth refers to any type of delivery that occurs between 20 and 37 wk of gestation. Women diagnosed with PTL due to complications associated with preterm birth have always been considered; however, its treatment remains a challenge. Non-pharmacological methods such as bed rest and reduced activity have been suggested but are not considered effective due to their low effectiveness. Therefore, the different drug has been suggested for women with the possibility of PTL, and each of these methods has different effects and is associated with complications that need to be considered. One of the first introduced drugs to these women was magnesium sulfate, which is known for its common maternal and fetal complications and there are conflicting reports about its effectiveness and high cost.

Currently, Tocolytic drugs are the first choice for women at risk of PTL to prevent uterine contractions. The findings of studies show that Nif which is a tocolytic with rapid absorption, tolerable and safe, has a good effect on reducing the risk of PTL (14). For example, the findings of the systematic review (15) in 2011 showed that in 26 selected studies with a population of 2179, Nif significantly reduced the risk of PTL for 7 days before 34 wk of pregnancy, and it was not significantly associated with remarkable complications compared to magnesium, and has shown better efficacy than other beta adrenergic agonist receptors. Several articles show that Nif had a good effect on controlling uterine contractions and delaying delivery (16, 17).

Another drug that has properties in common with Nif is sildenafil. SC inhibits the enzyme phosphodiesterase type 5. By selectively inhibiting phosphodiesterase type 5, it increases the concentration of C-guanosine monophosphate in vascular smooth muscle and also increases smooth muscle expansion. In studies conducted by Abd El-Naser and coworkers (18) and recent publication (19) have shown that Nif is one of the alternative drugs in inhibiting uterine contractions.

Fewer studies have studied the simultaneous effect of sildenafil and Nif in women at risk of PTL. The findings of one study (20) in vitro study showed that sildenafil could increase the effect of Nif by reducing intracellular calcium concentration with Nif. However, this study's generalization was questionable since it was performed outside the human body. The findings of another study (21) to evaluate the effect of sildenafil on myometrial muscles showed that uterine muscle expansion occurs immediately after taking sildenafil.

In the present study, we used a 25 mg vaginal sildenafil tablet every 8 hr. There is still no agreement as what dose is the effective dose of sildenafil in women at risk of PTL. To reduce systemic complications, we also used vaginal tablets that were not associated with significant complications in participants, and women tolerated them well. Studies also show that sildenafil is a safe drug that does not have a detrimental effect on the fetus and infant (7).

One of the strengths of the present study is that it can be tested and followed up with participants until delivery to evaluate the effect of the intervention. However, the present study has several limitations, including the relatively small volume of studied participants; complete blindness was not possible due to the type of intervention. Failure to evaluate the effect of sildenafil alone, and the effect of different doses of sildenafil and Nif on participants.

## 5. Conclusion 

The results of the study showed that Nif with SC is superior to Nif alone in women at risk of PTL due to increasing gestational age and better neonatal outcomes.

##  Acknowledgments

This study was funded by the Vice-Chancellor for Research and Technology of the Hamadan University of Medical Sciences, Hamadan, Iran (grand number: 140002281406). The authors thank Mr. Mohammad Faryadras for his benefit suggestion.

##  Conflict of Interest 

The authors declare that there is no conflict of interest.
